# Levator aponeurosis advancement with partial orbicularis oculi muscle resection for the treatment of Marin Amat syndrome with aponeurotic ptosis: two Case reports

**DOI:** 10.1080/23320885.2025.2451633

**Published:** 2025-01-13

**Authors:** Kaito Noguchi, Ikubun Osawa, Satomi Kurihara, Sho Yokoyama, Yosihiko Tanabe

**Affiliations:** a Department of Ophthalmology, Japan Community Healthcare Organization Chukyo Hospital, Nagoya-shi, Aichi, Japan; b Department of Ophthalmology, Iida Municipal Hospital, Iida-shi, Nagano, Japan; c Department of Plastic Surgery, Chubu Rosai Hospital, Nagoya-shi, Aichi, Japan

**Keywords:** Marin Amat syndrome, facial synkinesis, ophthalmic plastic and reconstructive surgery, aponeurotic ptosis

## Abstract

Marin Amat syndrome is a phenomenon in which eyelids close upon opening of the mouth during the recovery phase after facial nerve paralysis. In this report, we present two surgically treated cases of Marin Amat syndrome with aponeurotic ptosis. Case 1: A 66-year-old man had developed left Bell’s palsy a year prior to presentation and underwent rehabilitation at the Neurology Department of Japan Community Healthcare Organization Chukyo Hospital. He was subsequently referred to the Ophthalmology Department for left eyelid ptosis. Case 2: A 75-year-old man developed left Bell’s palsy more than 10 years prior to presentation and was referred to the Ophthalmology Department for left eyelid ptosis. Both patients had Marin Amat syndrome with aponeurotic ptosis. Levator aponeurosis advancement surgery and partial orbicularis oculi muscle resection were performed on the affected eyes. Both patients showed favorable postoperative outcomes. Simultaneous surgery involving levator aponeurosis advancement and partial orbicularis oculi muscle resection is effective for treating Marin Amat syndrome with aponeurotic ptosis.

## Introduction

Marin Amat [1] reported eyelid closure with mouth opening. This phenomenon was initially believed to be caused by an aberrant connection between the trigeminal and facial nerves. Because it is the opposite of the Marcus–Gunn phenomenon in that ptosis improves when the mouth is opened, it was termed the inverse Marcus–Gunn phenomenon. However, congenital cases of this phenomenon are extremely rare; most cases are acquired and caused by the aberrant regeneration of nerve fibers during the recovery phase after facial paralysis [2,3]. This acquired phenomenon is defined as Marin Amat syndrome (MAS) because it is an aberrant connection within the same nerve and is distinguished from the inverse Marcus–Gunn phenomenon.

MAS is a type of facial synkinesis characterized by the involuntary closure of the eyelid owing to the involuntary contraction of the orbicularis oculi muscle (OOM) in synchrony with the lower facial muscles during actions such as opening the mouth, protruding the lips, or puffing the cheeks. Therefore, MAS differs from the inverse Marcus–Gunn phenomenon, where ptosis is observed due to relaxation of the levator palpebrae superioris muscle [4].

Facial synkinesis is the involuntary movement of mimetic muscles associated with voluntary facial movements secondary to facial nerve injury. These involuntary movements have both cosmetic and functional consequences, leading to reduced quality of life [5].

Various mechanisms have been postulated to cause facial synkinesis, including nonspecific axonal regeneration and aberrant central nervous system adaptation. Currently, the leading theory involves nonspecific aberrant regeneration of nerves due to ineffective myelination and reorganization of neural networks during regrowth, resulting in ‘crosstalk’ between distal nerve fibers coupled with hypersensitization of the facial nucleus [6].

The diagnosis of MAS is often made based on characteristic clinical symptoms (① acquired, ② onset during the recovery phase after facial nerve paralysis, and ③ narrowing of the eyelid height associated with mouth opening), and electromyography (EMG) may be performed as a useful diagnostic test [7].

In this study, MAS was diagnosed based on the presence of characteristic clinical symptoms, and EMG was not performed. Additionally, because MAS often presents with aponeurotic ptosis, there is the potential risk of overlooking or underestimating it. Therefore, it is essential to enquire about the patient’s history, including facial nerve paralysis, in patients with eyelid ptosis [8].

Currently, effective treatments for MAS are limited. Local injection of botulinum toxin type A (BTX) [9,10] is the most common treatment, and biofeedback therapy [11] is occasionally used in combination with BTX injections. However, both BTX injections and biofeedback therapy are symptomatic therapies, and issues such as the need for repeated BTX injections and high treatment costs arise.

In a previous report of a patient with both MAS and acquired ptosis, levator aponeurosis advancement surgery improved the acquired ptosis but not the MAS [12].

In our report, we present two cases of MAS with aponeurotic ptosis, in which we performed both levator aponeurosis advancement surgery and OOM resection, with favorable outcomes.

This case series strictly adhered to the ethical principles outlined in the Declaration of Helsinki. In accordance with the Health Insurance Portability and Accountability Act (HIPAA), we affirm our commitment to safeguarding patient privacy and confidentiality. Protected health information has been handled with utmost care, and all necessary measures have been implemented to ensure compliance with HIPAA regulations. Patient identifiers have been appropriately anonymized and any potential identifiers have been removed to prevent the disclosure of sensitive information.

The patients consented in writing to the publication of the case.

## Case presentation

### Case 1

A 66-year-old man had been diagnosed with left Bell’s palsy 1 year prior to presentation and underwent rehabilitation for MAS at the Neurology Department of the Japan Community Healthcare Organization (JCHO) Chukyo Hospital. He subsequently developed aponeurotic ptosis in the left eye and was referred to the Ophthalmology Department of JCHO Chukyo Hospital.

**Figure 1.**
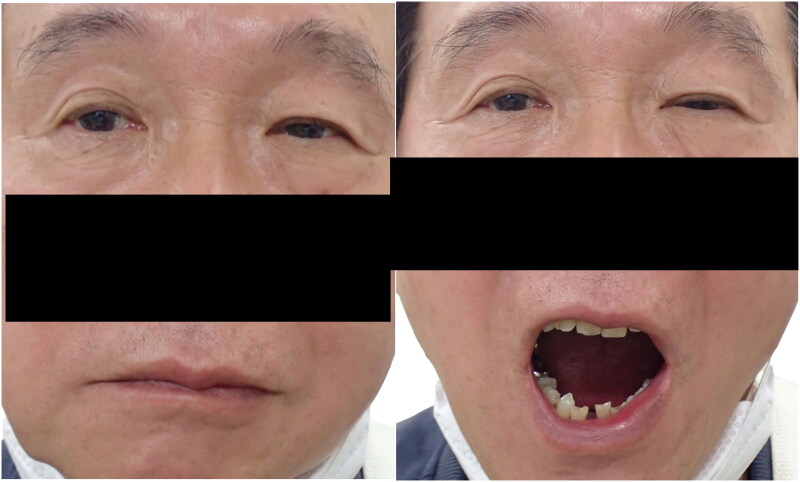
The preoperative photo for Patient 1 (Case 1) (left: mouth closing, right: full mouth opening). Ptosis of the left eye and closure of the left eyelid was observed during full mouth opening.

Initial examination revealed that the margin-reflex distance (MRD)-1 (distance from the upper eyelid margin to the central cornea) was 2.5 mm in the right eye and 1.5 mm in the left, the MRD-2 (distance from the lower eyelid margin to the central cornea) was 4.5 mm in the right eye and 3.5 mm in the left, and the levator function was 15 mm in the right eye and 14 mm in the left. During full mouth opening, the MRD-1 in the left eye decreased to 1 mm, and the MRD-2 decreased to 1.5 mm (Figure 1). The patient was able to lightly close their eyelids, and no paralytic lagophthalmos was observed, suggesting that OOM function had sufficiently recovered.

**Figure 2.**
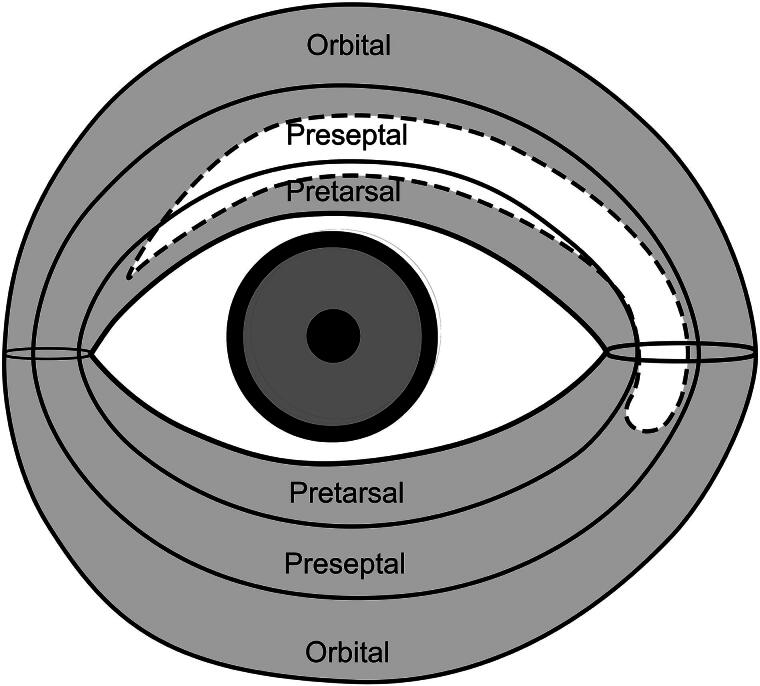
The three parts of the left eye orbicularis oculi muscle (orbital, preseptal, and pretarsal). The pretarsal and preseptal orbicularis oculi muscles of the upper eyelid and the preseptal orbicularis oculi muscle on the outer side of the lower eyelid were partially excised (area surrounded by the dotted line).

Levator aponeurosis advancement surgery was performed for aponeurotic ptosis, with a central 8 mm and lateral 10 mm width resection of the eyelid skin, along with 10 mm tucking of the levator aponeurosis using the white line as an anatomical landmark. Additionally, as part of the MAS treatment, the pretarsal and preseptal OOM of the upper eyelid and the preseptal OOM on the outer side of the lower eyelid were partially excised (Figure 2).

**Figure 3.**
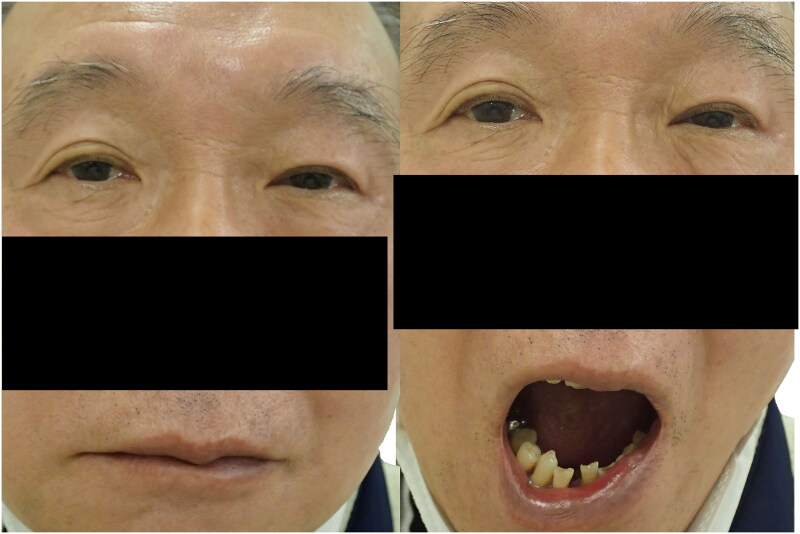
The postoperative photo for Patient 1 (Case 1) (left: mouth closing, right: full mouth opening). Ptosis of the left eye improved (left), and no eyelid closure of the left eye was observed during full mouth opening (right).

At the 6-month follow-up, the patient was very satisfied with the surgical results, the MRD-1 in the left eye had improved from 1.5 mm to 2.5 mm, and changes in the MRD-1 and MRD-2 during mouth opening had disappeared, indicating improvement in MAS (Figure 3). No complications, such as lagophthalmos and ectropion, were observed.

### Case2

A 75-year-old man had developed left Bell’s palsy more than 10 years prior to presentation. During treatment, he noticed drooping of the left eyelid, particularly during meals. Subsequently, he became aware of left eyelid ptosis not only when eating and he was referred to our department.

**Figure 4.**
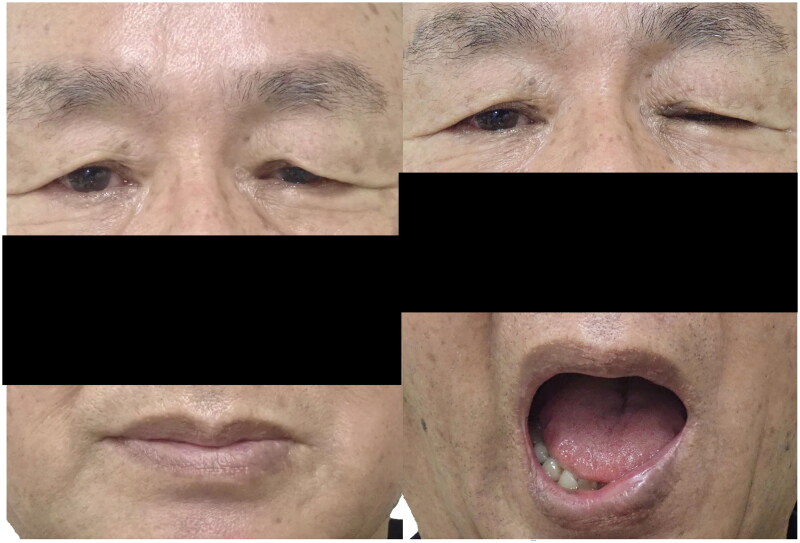
The preoperative photo for Patient 2 (Case2) (left: mouth closing, right: full mouth opening). Ptosis of the left eye and closure of the left eyelid was observed during full mouth opening.

On initial examination, the MRD-1 was 2 mm in the right eye and 1 mm in the left, MRD-2 was 4.5 mm in the right eye and 3 mm in the left, and levator function was 11 mm in the right eye and 10 mm in the left. During mouth opening, MRD-1 in the left eye decreased to 0 mm and MRD-2 decreased to 1 mm (Figure 4). The patient was able to lightly close their eyelids, and no paralytic lagophthalmos was observed, suggesting that OOM function had sufficiently recovered.

Levator aponeurosis advancement surgery was performed for aponeurotic ptosis, with a central 10 mm and lateral 15 mm width resection of the eyelid skin, along with a 10 mm tucking of the levator aponeurosis using the white line as an anatomical landmark. In addition, as part of the treatment for MAS, the OOM was excised, as in Case 1 (Figure 2).

**Figure 5.**
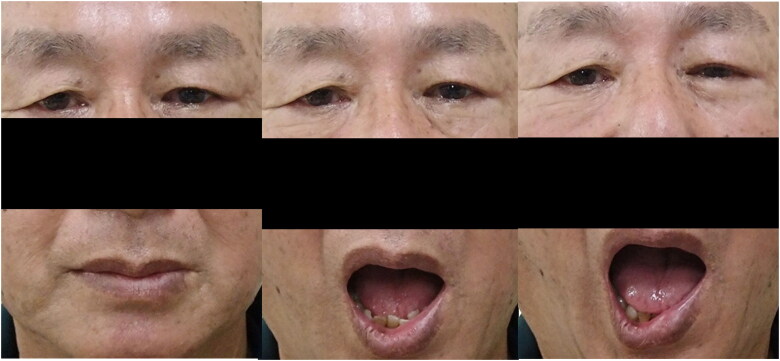
The postoperative photo for Patient 2 (Case 2) (left: mouth closing, middle: partial mouth opening, right: full mouth opening). Ptosis of the left eye improved (left). No eyelid closure was observed during partial mouth opening (Middle). Eyelid closure was observed during full mouth opening (right).

At the postoperative 6-month follow-up, the patient had a good course, with MRD-1 improving from 1 mm to 2.5 mm. The changes in MRD-1 and MRD-2 during partial mouth opening disappeared. The patient was no longer aware of the drooping of the left eyelid while eating. At full mouth opening, MRD-1 and MRD-2 decreased slightly to 1.5 mm and 2 mm, respectively (Figure 5). No complications, such as lagophthalmos and ectropion, were observed.

## Discussion

Treatment options for MAS include BTX injection [9,10] and biofeedback therapy using a mirror [11]. BTX injection is currently the most commonly used in clinical settings. It blocks the neuromuscular junction and reduces muscle strength, inducing a nonselective reduction in involuntary and voluntary muscle movements.

It is also used to treat blepharospasms and hemifacial spasms. For both efficacy and safety reasons, the low-dose administration of BTX injection is preferred [13]. However, BTX injection is a symptomatic treatment, which requires repeated administration and has associated high treatment costs.

The first-line treatment for blepharospasm, an involuntary contraction of the OOM, is also BTX injection. However, in cases in which it is ineffective or cannot be used, entire or partial OOM resection is sometimes performed [14–16]. Chapman et al. [15] reported OOM resection provides subjective benefit to patients with blepharospasm and decreases the long-term need for BTX injections in approximately 50% of these patients. Additionally, Gillum et al. [16] reported there were no cases of long-term lagophthalmos occurring due to the difficulty of complete removal of the OOM, however, the recurrences of blepharospasm were observed in some patients.

Because MAS is also characterized by involuntary contractions of the OOM, we hypothesized that it could also be improved by reducing involuntary contractions through OOM resection as shown in other papers [17–19]. In the present cases, owing to the coexistence of aponeurotic ptosis, extensive OOM resection was performed in conjunction with levator aponeurosis advancement surgery. BTX injections would have been considered if surgery did not yield sufficient MAS results. However, the surgery improved not only ptosis, but also MAS and subjective complaints.

Kikuchi et al. [12] reported a case of MAS with acquired ptosis in which levator aponeurosis advancement surgery was performed without OOM resection. Although the ptosis was successfully treated, the MAS did not resolve in this case. Additionally, Lai et al. [17] reported two cases of MAS without associated ptosis in which preseptal OOM resection led to the resolution of MAS. Also, Hsieh et al. [19] reported two cases of MAS selective myectomy and myotomy *in situ* in combination with levator plication improved MAS. Although OOM resection with levator aponeurosis advancement can be cause lagophthalmos, in our report, this combined approach resulted in improved MAS with no occurrence of lagophthalmos. Also, extensive OOM resection carries a risk of skin necrosis and hematoma; however, since the extent of OOM resection in this technique is limited, these risks are low. We consider partial OOM resection to be an effective treatment for MAS.

In our previous study [20], because BTX injections are usually administered in the pretarsal region, we performed a relatively extensive OOM resection in the pretarsal region for MAS. Additionally, when resecting the OOM to treat blepharospasm, as in our previous report, many surgeons target the OOM of the upper eyelid. However, in our current study, we not only resected the OOM at the site of skin resection, but also extended the resection below the lateral canthus. The involuntary contraction of the OOM due to MAS can extend to the lower eyelid; therefore, to reduce the involuntary contraction of the OOM in the lower eyelid, we resected this area. In our previous study, we only resected the OOM of the upper eyelid; however, a reduction in the MRD-2 during mouth opening was still observed postoperatively. In this report, particularly in Case 1, the change in the MRD-2 during mouth opening disappeared, suggesting the effectiveness of OOM resection below the lateral canthus. OOM resection below the lateral canthus can cause ectropion, but this can be prevented by avoiding excessive removal of the OOM and applying a pressure dressing for more than 12 h postoperatively. However, if ectropion is observed postoperatively, procedures such as a lateral tarsal strip may be required.

Finally, we discuss the possible reasons for the concomitant ptosis on the MAS-affected side. In this case, the coincidental occurrence of age-related changes on the MAS-affected side could not be entirely ruled out. However, the involuntary eyelid closure movement resisting the action of the levator palpe superioris muscle may have led to the stretching of the levator aponeurosis. Kikuchi et al. [12] have also pointed out this possibility, and it is plausible that MAS could cause the onset or progression of aponeurotic ptosis.

## Conclusion

We report two cases of MAS with aponeurotic ptosis in which we performed both levator aponeurosis advancement and partial OOM resection, resulting in favorable outcomes. Simultaneous surgery involving the advancement of the levator aponeurosis and partial OOM resection is considered an effective treatment for MAS accompanied by aponeurotic ptosis.

## Data Availability

Due to privacy concerns, the data are not publicly available but can be obtained from the corresponding author upon reasonable request.
